# SAMMate: a GUI tool for processing short read alignments in SAM/BAM format

**DOI:** 10.1186/1751-0473-6-2

**Published:** 2011-01-13

**Authors:** Guorong Xu, Nan Deng, Zhiyu Zhao, Thair Judeh, Erik Flemington, Dongxiao Zhu

**Affiliations:** 1 Department of Computer Science, University of New Orleans, 2000 Lakeshore Drive, New Orleans, LA 70148, USA; 2 Tulane Cancer Center and Tulane Health Science Center, 1430 Tulane Ave. New Orleans, LA 70112, USA; 3 The Research Institute for Children, Children's Hospital, 200 Henry Clay Ave. New Orleans, LA 70118, USA

## Abstract

**Background:**

Next Generation Sequencing (NGS) technology generates tens of millions of short reads for each DNA/RNA sample. A key step in NGS data analysis is the short read alignment of the generated sequences to a reference genome. Although storing alignment information in the Sequence Alignment/Map (SAM) or Binary SAM (BAM) format is now standard, biomedical researchers still have difficulty accessing this information.

**Results:**

We have developed a Graphical User Interface (GUI) software tool named SAMMate. SAMMate allows biomedical researchers to quickly process SAM/BAM files and is compatible with both single-end and paired-end sequencing technologies. SAMMate also automates some standard procedures in DNA-seq and RNA-seq data analysis. Using either standard or customized annotation files, SAMMate allows users to accurately calculate the short read coverage of genomic intervals. In particular, for RNA-seq data SAMMate can accurately calculate the gene expression abundance scores for customized genomic intervals using short reads originating from both exons and exon-exon junctions. Furthermore, SAMMate can quickly calculate a whole-genome signal map at base-wise resolution allowing researchers to solve an array of bioinformatics problems. Finally, SAMMate can export both a wiggle file for alignment visualization in the UCSC genome browser and an alignment statistics report. The biological impact of these features is demonstrated via several case studies that predict miRNA targets using short read alignment information files.

**Conclusions:**

With just a few mouse clicks, SAMMate will provide biomedical researchers easy access to important alignment information stored in SAM/BAM files. Our software is constantly updated and will greatly facilitate the downstream analysis of NGS data. Both the source code and the GUI executable are freely available under the GNU General Public License at http://sammate.sourceforge.net.

## Background

Next generation deep sequencing technology has recently emerged as a promising tool to simultaneously and accurately quantify DNA/RNA abundance on the genomic scale [1]. The alignment of tens of millions of short reads to a reference genome is a central step for subsequent data analysis. A variety of short read alignment tools are currently available that implement fast, efficient and accurate short read alignments against larger reference genomes. Some commonly used alignment tools include MAQ [2], Novoalign http://www.novocraft.com/, Bowtie [3], rMap [4] and RMAP [5]. Many of these tools output the alignment results in the Sequence Alignment/Map (SAM) and Binary SAM (BAM) formats [6], which are widely considered the *de facto *standards for storing and transferring short read alignment results. Correspondingly, there are a number of open source software programs that process the alignment results stored in SAM/BAM files. For example, SAMtools http://samtools.sourceforge.net/ provides various utilities for manipulating alignments in SAM/BAM files including sorting, merging, indexing and generating alignments in a base-wise format [6]. Another program, DNAA http://dnaa.sourceforge.net/, calculates alignment statistics, detects structural variation and simulates short-read data. For genome alignment visualization software, one may use GenomeView http://genomeview.sourceforge.net/ or IGV http://www.broadinstitute.org/igv. These programs have been very useful in analyzing NGS data and visualizing alignment results.

Nevertheless, a plethora of frequently needed genomic information stored in SAM/BAM files remains hidden from biomedical researchers. For example, one can calculate from SAM/BAM files the mRNA abundance scores from RNA-seq data [7] that are used to detect differential expression. Furthermore, SAM/BAM files may contain a base-wise signal map from RNA-seq [8], ChIP-seq [9] or Methyl-seq data [10] that is used to detect gene expression abundance, transcription factor binding sites or DNA methylation sites. SAM/BAM files may also store data about the short read coverage depth visualization needed to examine the gene or transcriptome structure alteration. Finally, SAM/BAM files contain alignment statistics needed to evaluate the performance of different alignments. In the past it has been quite tedious for biomedical researchers to access this rich data stored in SAM/BAM files. These significant hurdles have prevented biomedical researchers from fully exploiting the benefits of NGS technology. Therefore, SAMMate with its user friendly interface fulfills a critical role by giving biomedical researchers easy access to essential data.

**Figure 1:**
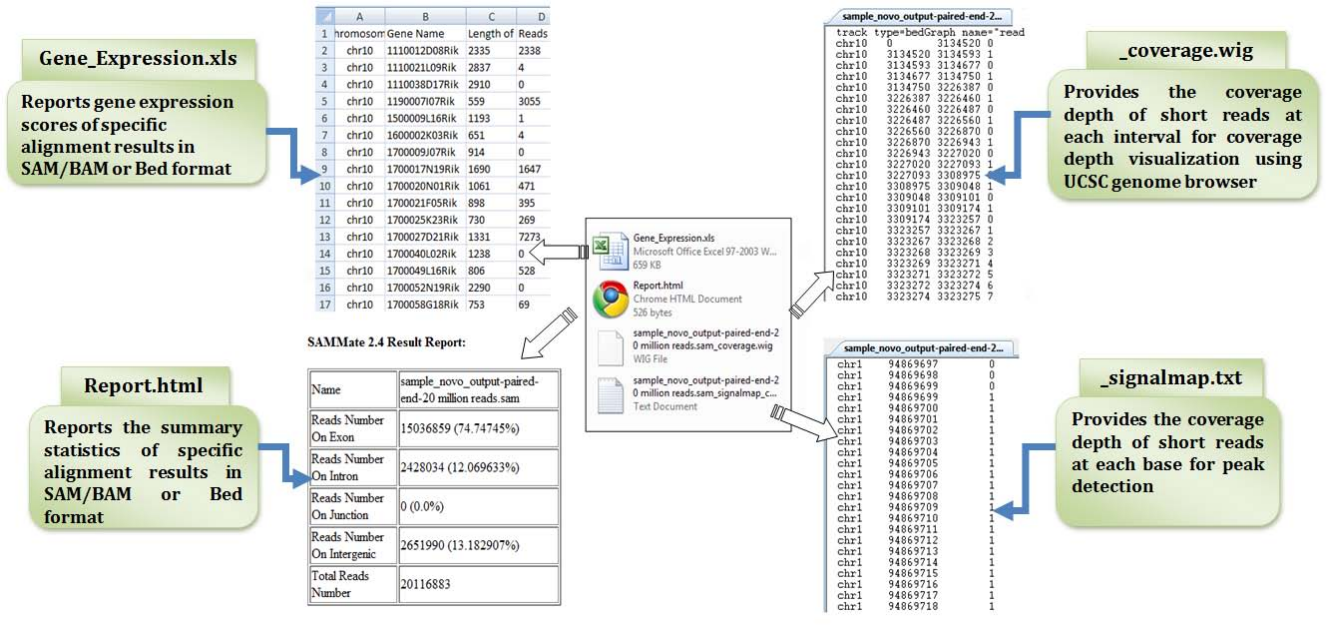
**The Four Key Features of SAMMate**. A schematic diagram of the four key features of SAMMate. (1) Fast calculation of gene expression RPKM scores combining exon reads and junction reads. (2) Fast calculation of whole-genome base-wise signal map. (3) Generation of wiggle files for short read alignment visualization. (4) Generation of an alignment statistics report.

In this article we introduce SAMMate. SAMMate is a GUI software tool that allows biomedical researchers to easily access essential information stored in SAM/BAM files. A detailed documentation and a quick walkthrough are available at SAMMate's homepage http://sammate.sourceforge.net. SAMMate possesses the following key features (Figure 1): (1) For RNA-seq alignment SAMMate uses short reads originating from both exons and exon-exon junctions to calculate gene expression scores. SAMMate's versatility allows biomedical researchers to combine the output from an exon alignment program, such as Novoalign http://www.novocraft.com/, with the output of a splice junction analysis program, such as TopHat [11]. This intuitive combination results in a more accurate estimation of gene expression abundance scores. (2). Using SAM/BAM files generated from short read alignments, SAMMate implements an efficient and fast algorithm to calculate a base-wise signal map. For large SAM/BAM files with more than 6 million records, it only takes approximately one minute on a standard desktop or laptop computer to generate the base-wise signal map that falls between the customized intervals. (3). SAMMate also exports a wiggle file for visualization of alignment results on the UCSC genome browser. (4). Lastly, SAMMate exports an alignment statistics report. In addition, SAMMate has nice utilities for manipulating SAM/BAM files that include merging and sorting. We have designed several case studies in the context of miRNA-155 target prediction to demonstrate the key features of SAMMate since these features are essential for solving a wide range of biological problems using NGS data.

## Implementation

### User Interface Overview

The central pillar of SAMMate's design philosophy is ease of use for biomedical researchers. A user friendly interface and portability are essential to this goal. To achieve portability in a cross-platform environment, we have implemented SAMMate in Java. As such, our GUI tool SAMMate runs almost identically on the Windows, Mac and UNIX/Linux operating systems. A key advantage of using Java for software development lies in its independence from processor architectures and operating systems. Java achieves this independence via its use of a common executing engine (also known as the virtual machine) that has been implemented across different platforms. A compiler with a set of standard libraries has also been implemented for various hardware and operating systems. The only extra software needed by SAMMate is the freely available Java Runtime Environment (JRE), which is already available on most operating systems.

**Figure 2:**
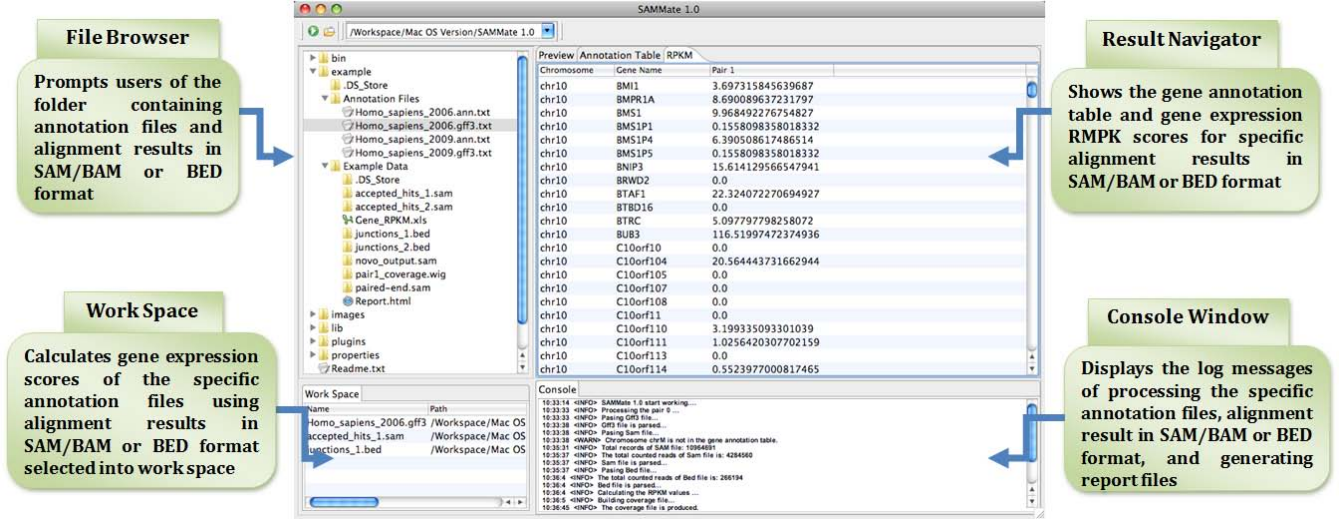
**SAMMate Graphical User Interface**. A snapshot of the four panels of the SAMMate Graphical User Interface: File Browser, Workspace, Result Navigator and Console Window

For the interface's building components, we exploited the latest and best Java technology currently available from Eclipse http://www.eclipse.org/. SAMMate makes use of Eclipse's Standard Widget Toolkit (SWT) and JFace. SWT is a low-level GUI tool kit comparable in concept to the AWT package present in Java. SWT possesses a look and feel of the native operating system. SWT also combines the best elements of both AWT and Swing implementations and overcomes the limitations found in AWT and Swing. As for JFace, it is a set of enhanced components and utility services that makes building GUIs with SWT easier. These components aided in the development of a sharp and neat user interface greatly benefiting biomedical researchers who now have an alternative to command-line interfaces. SAMMate's advantages go even beyond its aesthetic appeal. In addition to SAMMate's cross-platform compatibility, SAMMate is also very easy to use. With a few mouse clicks, users can easily view, control and manipulate multiple utilities all at once. SAMMate also spares biomedical researchers from the technicalities of compiling a tool from source code. Instead, researchers can focus on what matters: gaining biological insight from NGS data. The entire SAMMate interface is partitioned into four panels (Figure 2): (1) *File Browser *prompts users for SAM/BAM and/or BED files to be used as input. (2) *Work Space *calculates gene expression scores using annotation files and the alignment results of the input files. (3) *Result Navigator *displays the gene annotation table and the gene expression scores of a specific alignment result in SAM/BAM or BED format. (4) *Console Table *shows the log messages of processing the annotation files and the alignment results in SAM/BAM or BED format. It can also generate report files.

### Software Architecture

**Figure 3:**
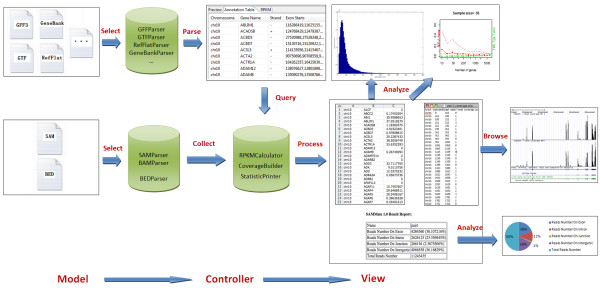
**SAMMate Software Architecture**. A snapshot of the SAMMate software architecture.

We now provide a brief description of the software architecture to enlighten users about key SAMMate modules and their interconnections. The architecture of SAMMate follows the standard Model-View-Controller (MVC), a common architectural pattern used in software engineering. The MVC approach decomposes the problem into input data (model), presentation of the data (view) and business logic (controller) (Figure 3). The basic software architecture has three components: *UI *package (Presentation of the data processing module), *Calculator *package (Business logic processing module) and *Alignment *package (Input data processing module). The advantages of adopting the MVC approach are malleability, modularity, reusability, flexibility and extensibility.

SAMMate's reusability is very robust as developers can easily reuse existing classes by using the *new *method to create an instance of a class. SAMMate is also very extensible. If a user wishes to expand upon a component, the user may simply use the *extends *keyword to inherit the methods and properties of the desired class. Developers may also conveniently add classes to implement new features. For example, developers may add a new parser to process a gene annotation file in a new format by adding a class to the Alignment package. Furthermore, a developer may add a new tab in the UI package to display results in the result navigator window at their own preference. One other important feature of SAMMate is its configurability. For example, SAMMate allows users to customize the chromosome names in output files to their own preferred chromosome names by manipulating the configuration file "chromosomesMap.txt". In summary, the SAMMate software architecture implements a number of Applied Programming Interfaces (API's) so that other software developers may easily extend and build more utilities.

## Results

### Overview

Using the standard reference genome annotation files, SAMMate allows users to accurately calculate the gene expression abundance scores for all annotated genes using RNA-seq data. SAMMate can also calculate the abundance scores of customized genomic intervals. In particular, SAMMate is able to use short reads originating from both exons and exon-exon junctions to accurately calculate gene expression scores. We used *in vitro *3'-UTR analysis to illustrate the superior reporting capability of SAMMate's gene expression scores compared with other competing software like TopHat [11]. 3'-UTR assays are a widely used low-throughput and accurate method to quantify microRNA dependent post-transcriptional regulation. Fold change is a frequently used metric, especially for biologists, to access the magnitude of gene regulation, either up-regulation or down-regulation. Therefore, we expect a better method to quantify gene expression abundance using RNA-seq data can be determined by comparing the resulting fold changes to those calculated from more accurate low-throughput experiment, such as 3'-UTR assay. SAMMate can also generate wiggle files for visualization in the UCSC genome browser as well as an alignment statistics report. It is also compatible with both single-end and paired-end sequencing technologies. SAMMate is freely available under the GNU General Public License. The key features provided by SAMMate are outlined in Figure 1 and are thoroughly illustrated via the case studies below. The SAMMate software manual was also provided as Additional file 1.

### Key Feature: Calculating genomic feature abundance scores

Ideally, transcriptome characterization and quantification should be done on the isoform level. However, many existing approaches that quantify transcript abundance on the isoform level depend upon stringent assumptions such as *a priori *known isoform structures and suffer from identifiability problems. Moreover, the accuracy of these algorithms for high throughput studies is in doubt. It is also not known how sensitive these algorithms are to error-prone isoform annotation databases. Many existing approaches [6,12,13] have proved their merit as pilot studies. However, they were only validated using RT-PCR for a limited number of genes with simple and identifiable isoform transcript structures. With the aforementioned in mind, transcript abundance quantification on the gene level remains one of the most demanded outputs from high throughput molecular profiling experiments such as microarray and NGS. The latter platform is much more sensitive in detecting low level gene expression and provides a much broader dynamic range of expression quantification. Taking gene expression profiling using Illumina Genome Analyzer as an example, Read Per Kilobase of exon model per million Mapped reads (RPKM) was used to score gene expression abundance. The values obtained can be interpreted as the number of copies of each transcript in the living cell where the average length of transcripts is 1KB [8]. The RPKM scores can range from *<*0.01 to *>*10,000. There are now unprecedented and unparalleled opportunities to detect novel transcripts with ultra-low or ultra-high abundance.

**Figure 4:**
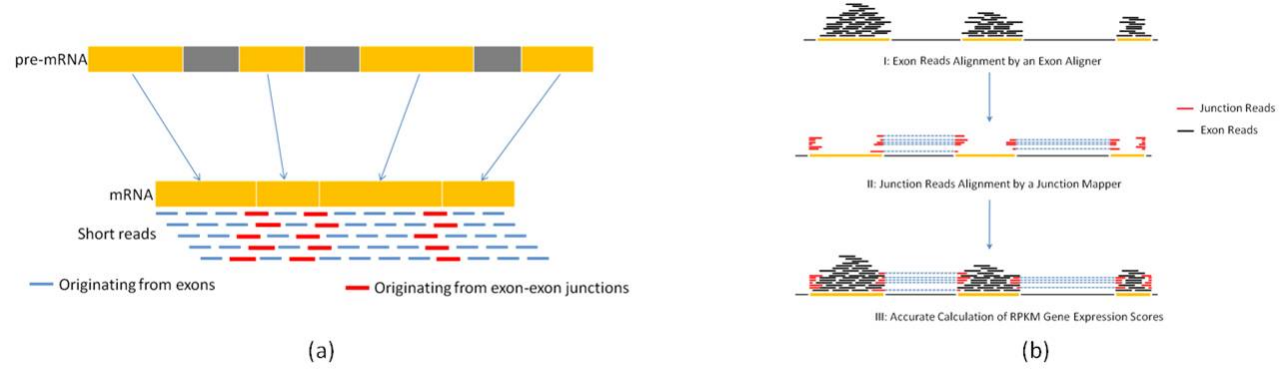
**Combination of Exon Reads with Junction Reads to Accurately Calculate Gene Expression RPKM Scores**. (a) A unique challenge for researchers working with RNA-seq data. The junction reads (red) fail to map back to the reference genome because exons are separated by introns. (b) A demonstration of the ideas of combing exon reads (black) and junction reads (red) to calculate gene expression RPKM scores.

A unique challenge for researchers working with RNA-seq data is short reads originating from exon-exon junctions (around 10%). These short reads fail to map back to the reference genome since the exons are separated by introns (Figure 4a). The millions of unmapped short reads originating from exon-exon junctions, denoted as Initially Unmapped Reads (IUM's), need to be accounted for when calculating RPKM scores [11]. Unfortunately, most alignment tools only map the short reads originating from exons completely ignoring IUMs in the process. Hereinafter, we denote such aligners as "exon aligner". To address the limitations of "exon aligners", ERANGE [8], TopHat [11] and rSeq [4] are among the recently developed approaches to map IUM's originating from exon-exon junctions back to individual genes. ERANGE uses a union of known and novel junctions while TopHat *de novo *assembles IUM's using a module in Maq [2]. Hereinafter, we denote an aligner of this type as "junction mapper". Thus, there are now two types of aligners that complement each other.

Performance-wise, aligners vary vastly in accuracy as well as the underlying algorithms used. It is highly desirable for RNA-seq data analysis to allow users the freedom to choose and combine a pair of their favorite exon aligner and junction mapper to estimate gene expression scores. SAMMate fulfills this role by calculating and exporting a gene expression score matrix using a user-defined combination of an exon aligner and a junction mapper (Figure 4b). SAMMate then calculates the gene expression RPKM or FPKM score for gene *i*, ℛ as ${ℛ}_{i}=\frac{10^{9}(C_{i}^{A}+C_{i}^{B})}{NL_{i}}$ where *i *represents the gene index. $C_{i}^{A}$ is the short read counts uniquely mapped to exons using an exon aligner (e.g. Novoalign), and $C_{i}^{B}$ is the IUM short read counts uniquely mapped to the exon-exon junctions using a junction mapper (e.g. TopHat). *N *represents all uniquely mapped read counts in a cell extract sample, and *L_i _*is the summation of the exon lengths. Thus, SAMMate combines short reads mapped to exons (e.g. available in SAM/BAM format) and to exon-exon junctions (e.g. available in BED format) to accurately estimate gene expression scores (Figure 4b). SAMMate can also take many pairs of SAM(BAM)/BED files simultaneously, one for each cell sample, to calculate a Microsoft EXCEL compatible gene expression matrix. In this matrix rows correspond to genes or the customized genome coordinate intervals, and columns correspond to different cell samples. It must be noted that SAMMate is more flexible and accurate than other software, such as TopHat [11], that also export the gene expression scores. We validate our claim using experimental data obtained from 3'-UTR assay as a case study shown below. SAMMate's reporting utility for gene expression abundance score is also quite versatile as this utility is not limited to the annotated genes. In fact, SAMMate calculates genomic feature abundance scores for any user-defined genomic intervals. This utility dramatically simplifies the technical barriers for discovering novel genes.

#### Algorithmic and computational contributions

**Figure 5:**
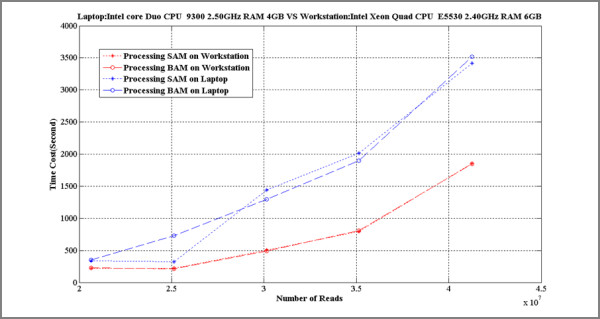
**Test of Scalability**. The computational scalability of SAMMate in processing an increasing array of SAM/BAM files were examined by accessing the system time needed to calculate the genomic feature abundance scores. One typical desktop (Intel Xeon Quad CPU E5530 2.40GHz RAM 6GB) and one typical laptop (Intel core Duo CPU 9300 2.50GHz RAM 4GB) were used for this experiment.

SAMMate uses a novel mapping and sorting strategy to create an ultrafast, efficient calculation of gene expression abundance scores as well as generating wiggle files for visualization. To parse gene annotation files, we used a highly efficient Java data structure, Hash Table, to store gene lists for each chromosome. Since the number of genes in each chromosome is at least one order of magnitude smaller than ten thousand, the memory complexity of storing a gene list for each chromosome using a Hash Table is moderate. Furthermore, the time complexity to search for a gene element in a Hash Table is O(1). Our approach is also very advantageous for subsequent calculations of gene expression scores, generation of wiggle files and displaying results in the SAMMate navigator windows. In parsing SAM/BAM files and BED files, SAMMate makes a number of reasonable compromises to achieve computational efficiency. According to the current statistics summary of the *Homo sapiens *genome (Build 37 version 1), the length of a mammalian chromosome ranges from 50 Millon bp to 250 Millon bp. Obviously, it is not economical to allocate a huge memory block to save such a long coordinate. SAMMate remedies this situation by storing only the start position of the short reads in a sorted list by chromosome resulting in an average length of less than 1 Million for each lane at the current throughput (number of short reads in each lane). It is quite scalable even if the throughput for each lane were to increase in the near future. Figure 5 illustrates the scalability of SAMMate processing an array of SAM/BAM files of increasing sizes.

#### Biological case study: Comparing gene expression scores generated using SAMMate, TopHat and Novoalign to predict miRNA targets

We have studied a pair of control and treatment transcriptomes. The control transcriptome was derived from the wild-type MutuI cell line while the treatment transcriptome was derived from the miRNA-155 retrovirally transduced MutuI cell line [14]. MicroRNAs plays pivotal roles in controlling normal and pathology associated cellular processes. Moreover, the importance of miRNA dysregulation in cancer is well known and a number of tumor promoting miRNA's have been identified. As a member of this class of microRNAs, miR-155 is implicated in lymphomagenesis and a wide array of nonlymphoid tumors including breast, colon, and lung. Despite the strong evidence for miRNA-155 as an oncogene, the underlying pathological mechanisms remain unclear, possibly due to limited knowledge of miRNA-155 targets and how these targets are involved in tumorigenesis [15,16]. Both transcriptomes were profiled using the Illumina Genome Analyzer II platform with a 50-mer in read length. For each transcriptome, two biological replicates were used. Each biological replicate has 2 to 4 technical replicates nested within it. Each technical replicate of the transcriptome (a single lane in each instrument run) contains around 6 to 12 million short reads. This NGS data set is available at the National Center for Biotechnology Information (NCBI) Short Read Achive (SRI) website http://www.ncbi.nlm.nih.gov/sra with access code SRA011001. We aligned the short reads generated from each transcriptome to the reference human genome (Build 37 version 1) using Novoalign, Bowtie and TopHat respectively allowing up to two mismatches. The alignment information of the exons and exon-exon junctions were stored in SAM/BAM and BED formats. Our biological goal is to predict a list of miRNA-155 direct targets on the genomic scale. Accurate calculation of the gene expression scores is a central problem for achieving this goal since down-regulated genes are likely to be potential miRNA-155 direct targets.

**Figure 6:**
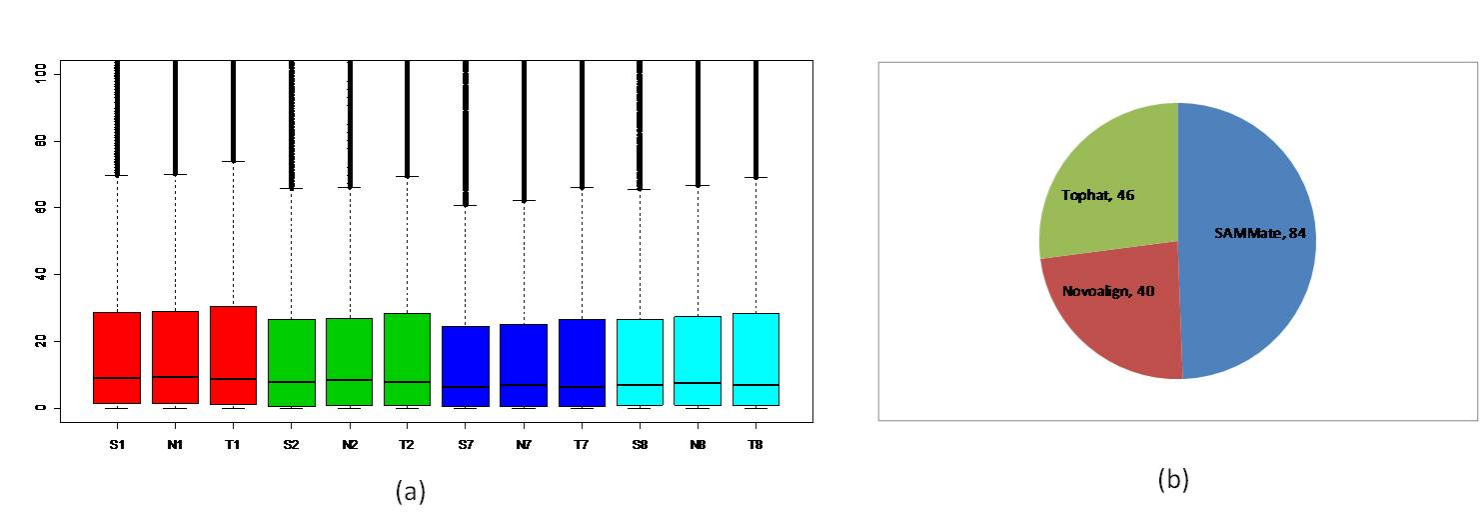
**Comparison of RPKM Gene Expression Scores Reported by SAMMate, Novoalign and Tophat**. (a) Comparison of the overall RPKM distributions calculated by different methods. 'S' stands for SAMMate, 'N' stands for Novoalign, and 'T' stands for Tophat. '1' and '2' represent two biological replicates of the wild type transcriptome (control). '7' and '8' represent two biological replicates of the miRNA-155 transfected transciptome (treatment). For example, 'S1' stands for the RPKM score distribution calculated from the first biological replicate transcriptome of wild type Mutu I cell line using SAMMate. There are no significant differences both visually and statistically. (b) Piechart of percentages of gene fold changes calculated by each tool that are closest to the 3'-UTR experimental results. SAMMate is superior to the other competing tools.

We used SAMMate to calculate RPKM gene expression scores for each transcriptome and compared these results with RPKM gene expression score distributions calculated using TopHat and Novoalign alone. Figure 6(a) plots truncated boxplots for RPKM gene expression score distributions calculated by SAMMate ('S'), Novoalign ('N') and TopHat ('T'), respectively. No significant difference, either visually or statistically, was observed between distributions of all annotated gene expression RPKM scores. We then proceeded to compare the accuracy of the RPKM score reporting capability for the three tools using a selected set of 170 genes for which a 3'-UTR assay was performed for each gene. To make the RPKM scores calculated from the *in vivo *whole-genome sequencing experiments comparable with the fluorescent intensity scores calculated from the *in vitro *3'-UTR assay, we used the expression fold change method between treatment (miRNA-155 transduced) and control (wild type) for each gene. Thus, the best tool at reporting gene expression possesses fold changes that are closest to those calculated from 3'-UTR assays. Figure 6b shows SAMMate is more accurate than the competitors for gene expression score calculation in 84 out of 170 genes (49%). The numbers for TopHat and Novoalign are 46 (27%) and 40 (24%), respectively (Figure 6b). Our validation study using 3'-UTR analysis provides compelling evidence that SAMMate exports gene expression RPKM scores more accurately than its competitors.

### Key Feature: Generating signal map for peak detection

A signal map is a frequently demanded data format for NGS data analysis. In a signal map file, alignment results are represented in the per-base "pileup" format. In this format the single nucleotide short read coverage depth is calculated whereas the whole genome coverage is provided as a vector of integers with length 3.2 × 10^9^. A signal map is a common input for a number of frequently performed sequential analyses to detect a wide range of genomic features. For ChIP-seq and Methyl-seq data, significant peaks in a signal map may indicate potential transcription factor binding sites and DNA methylation sites, respectively. For DNA-seq data, significant change points in the signal map might indicate a true copy number change, which is often a hallmark of cancer [17].

Although generating a signal map may appear to be straightforward, the computational issues are non-trivial. In the worst-case scenario, the computational complexity for brute force parsing is to the order of 10^17^. Due to the huge data size of NGS, an efficient algorithm must be implemented to make generating a signal map feasible on a desktop computer. SAMMate's algorithm for signal map generation takes only one minute or so on a standard desktop or laptop computer (Intel core Duo CPU 9300 2.50GHz RAM 4GB) for a SAM file that includes more than 10 million records. We also generated a human signal map using SAMtools [6], a popular command-line tool to generate signal maps. We compared its performance (using UNIX command 'time') with the performance of SAMMate. Table 1 clearly indicates that SAMMate takes much less system time to generate a human signal map than SAMtools. Factoring in SAMMate's user friendly interface, the potential time saved by researchers is quite significant.

**Table 1:** Comparison between SAMMate and SAMtools

Running Time Comparison of SAMMate and SAMtool (SAM File: 6 *10^6^ records, 1.03GB)				
**Software**	**Hardware Platform**	**SAM File Parsing Time**	**Sorting Time**	**Total Time**

SAMMate	Intel core Duo CPU 2.26 GHz RAM 2GB	60s	43s	132s

SAMtools	Intel core Duo CPU 2.26 GHz RAM 2GB	93s	210s	310s

SAMMate	Intel core Duo CPU 9300 2.50GHz RAM 4GB	36s	25s	72s

SAMtools	Intel core Duo CPU 9300 2.50GHz RAM 4GB	57s	122s	183.8s

SAMMate and SAMtools were compared for the amount of system time needed to generate one human signal map file using a SAM file. Two typical laptops were used for this comparison.

#### Algorithmic and computational contributions

In calculating a signal map, SAMMate uses an optimized merge sort algorithm to sort the list of the start position of short reads. It was implemented using the "Collections" class in the JRE system library. The optimized merge sort algorithm has *nlog*(*n*) time complexity. For nearly sorted lists, the optimized merge sort algorithm runs substantially faster. Although a highly optimized quicksort algorithm is generally considered to be faster than a merge sort algorithm, the quick sort algorithm is not stable and provides no guarantee of achieving a time complexity anywhere near *nlog*(*n*). The optimized merge sort algorithm also does not reorder equal elements yielding another significant advantage. A huge decrease in time needed is obtained as repeatedly sorting the same list (many short reads in one lane may have the same start position) is no longer done.

#### Biological case study: Genome-wide change-point analysis to identify potential miRNA targets

Other than down-regulation at the whole gene locus level, another characteristic response of potential miRNA-155 targets is only visible from the signal map: an abrupt drop-out of the base-wise coverage in the 3-prime end. For this case study, we have the same biological goal as the previous case study, i.e., to predict miRNA-155 targets. We used SAMMate to calculate a signal map for each biological replicate. We then applied a genome-wide change point analysis to all of the annotated 3'-UTR regions to identify potential miRNA targets. We also compared the predicted miRNA-155 targets generated using

RPKM gene expression scores (previous case study) with the ones generated using signal maps (this case study). We hope these two orthogonal and complementary case studies that share the same biological goal will be more than adequate to demonstrate the robustness and key features of SAMMate. For the sake of completeness, we briefly introduce the change point analysis method that we have applied [17]. For each annotated 3'-UTR, we test the null hypothesis of the equal mean and variance parameters in the sequence of base-wise signal. The alternative hypothesis is the unknown number of change points' position exists. For statistical analysis we applied a Mean and Variance Change point Model (MVCM) based approach [17]s. The input of the change point analysis algorithm is two sets of signal map files generated by SAMMate where each set is a series of replicates of a genome sample. In each signal map file, there are over 249*; *000 bases. The output for each annotated 3'-UTR is a list of change point positions sorted by ascending order according to their Schwarz Information Criterion (SIC) values [17]. The 3'-UTR coordinates were determined by the human genome annotation file (version hg19) with over 34*; *000 annotated 3'-UTR's in total.

**Figure 7:**
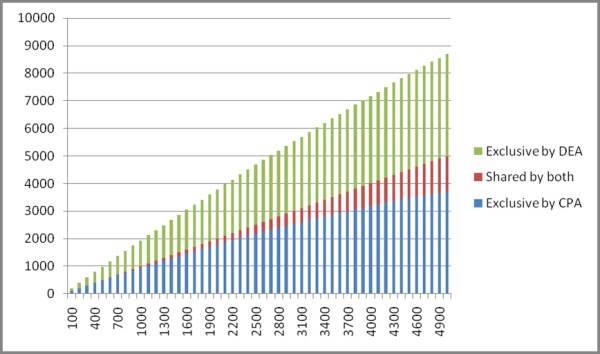
**Comparison of Differential Expression Analysis (DEA) and Change Point Analysis (CPA) in Prediction of miRNA-155 Targets**. (a). The horizontal axis represents gene cut-off's while the vertical axis represents the number of genes called by DEA, CPA or both. (b). Validation analysis of the prediction results in (a) against the set of 170 genes where the *in vitro *3'-UTR assays were performed. (c). Examples of predicted targets with detected change points in the 3'-UTR end.

We calculated the difference between the base-wise average for each set of signal map files, representing a single differential signal map for wild-type MutuI cell line and miRNA-155 transfected MutuI cell line. It was then followed by a calculation of the SIC values for each annotated 3'-UTR. The change point analysis on the differential signal map between wild type and miRNA-155 transduced MutuI cells was parallelized for multi-threading with OpenMP to overcome computational challenges. Figure 7a presents a comparison of the ranked gene lists called by Differential Expression Analysis (DEA) and Change-Point Analysis (CPA). The horizontal axis represents the gene number cut-offs starting from 100 to 5000. Three sets of genes are shown at each cut-off: putative miRNA targets predicted exclusively by CPA, exclusively by DEA, and by both DEA and CPA. Both methods for miRNA target prediction are able to identify a common set of genes, and each method has a unique gene list. Figure 7b shows the validation studies of the putative miRNA targets against the set of 170 genes for which 3'-UTR assays were performed. Figure 7a and Figure 7b are consistent with one another. The results support the notion that a combination of DEA and CPA is able to predict a comprehensive and a conserved list of miRNA targets. SAMMate provided the essential input, i.e. gene expression scores and signal maps, for both DEA and CPA as reported in the two case studies. In summary, SAMMate is a highly valuable tool to compare two orthogonal and complementary approaches to detect gene structure alterations.

### Key Feature: Generating wiggle files for visualization

**Figure 8:**
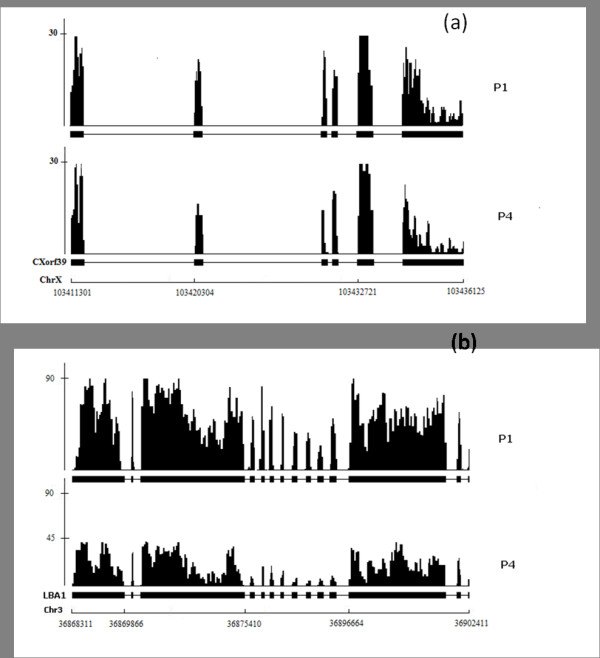
**Visualization of Gene Structure Variation**. Two typical examples were shown: (1) Gene CXorf39 was called by the Change Point Analysis as a potential miRNA-155 target due to it's abrupt read dropout on the 3'-UTR end. (2) Gene LBA1 was called by the Differential Expression Analysis as a potential miRNA-155 target due to the overall read coverage decrease in codon region.

Biomedical researchers also need to visualize the alignment results stored in SAM files in order to examine possible gene structure alterations between case and control studies. For example, shortened 3'-UTR's in cancer cells are reflected as an abrupt dropout of the short read coverage. This visualization need is addressed by another key feature of SAMMate. SAMMate can take the alignment results stored in SAM files and export the genome information to wiggle (.wig) files where the wiggle format is compatible with the UCSC genome browser and other browsers used for visualization. This feature will allow biomedical researchers to visually check the alignment quality of selected genes in selected genomic regions. For the miRNA-155 target prediction research, Figure 8 presents two typical scenarios: the left and right panels show the alignment results in the pile-up format for gene CXorf39 on chromosome X and gene LBA1 on chromosome 3, respectively. Figure 8a indicates no overall expression change in the codon regions, but a significant dropout in the 3'-UTR region occurs. On the contrary, Figure 8b shows no significant difference in the 3'-UTR region but a significant difference in the codon region instead. These two examples demonstrate SAMMate's ability to generate wiggle files for biomedical researchers allowing them to visually look for possible gene structure alterations. While there are a number of existing alignment visualization software (e.g. [18,19]), these systems do not allow many annotation tracks in parallel, which is the deterministic feature for knowledge discovery.

### Key Feature: Generating an alignment report

Short read alignment statistics provide indispensable resources to examine the alignment quality as well as compare alignment results. SAMMate calculates and exports a number of alignment statistics including the percentage of uniquely mapped short reads and the percentage of short reads mapped to intergenic, exonic and intronic regions.

## Conclusion

We have implemented a GUI software to allow biomedical researchers to parse, process and integrate alignment information found in SAM files. With this tool biomedical researchers are able to calculate gene expression scores using either standard or customized annotations. They are also able to visualize and compare alignment results with great ease. These utilities and their biological impact are adequately demonstrated via the case studies of miRNA target prediction. The biological applications of SAMMate, however, are not limited to miRNA target prediction alone. In fact, SAMMate applies to any biological problem whose solution depends on the gene expression abundance score and base-wise short read coverage signal. SAMMate is also highly modular and extensible providing a programmer friendly interface for ease of updates and the incorporation of contributions from the community. Our tool will greatly facilitate the downstream analysis of genomic sequencing data.

## Competing interests

The authors declare that they have no competing interests.

## Authors' contributions

GX designed and implemented the graphical user interface using Java and Eclipse. ND, ZZ, DZ contributed to both software design and data analysis. EF provided biological inputs and experimental data for analysis. DZ designed the study, oversaw the project and wrote the manuscript. All of the authors listed have read, revised and agreed to the manuscript.

## Supplementary Material

Additional file 1 **SAMMate Software Manual**. A detailed demonstration of the key features of SAMMate and some user tips. Click here for file
