# Supplementary material for: SAMMate: a GUI tool for processing short read alignments in SAM/BAM format
Source: Source Code Biol Med. 2011 Jan 13;6:2. doi: 10.1186/1751-0473-6-2 (PMC3027120; doi:10.1186/1751-0473-6-2)
Supplement: Additional file 1 — SAMMate Software Manual. A detailed demonstration of the key features of SAMMate and some user tips. [file 1751-0473-6-2-S1.PDF]

SAMMate

Computational Biology Research Group
Department of Computer Science
University of New Orleans

January 2011

[MANUAL]

SAMMate allows biomedical researchers to quickly process SAM/BAM files. This software is constantly updated and will greatly facilitate the downstream analysis of NGS data in the SAM/BAM format.
Both the source code and the GUI executable are freely available at http://SAMMate.sourceforge.net.

SAMMate Manual

Copyright © 2009-2011 Computational Biology Group@UNO Computer Science. All rights reserved.

Abstract

Welcome to the *SAMMate* Manual. Here you will find information on how to install and configure the application. It is a step-by-step, task-oriented guide for configuring *SAMMate* on your system.

License

This document is maintained by the Computational Biology Group at UNO Computer Science and is freely available under the GNU General Public License.

SAMMate is free software; you can redistribute it and/or modify it under the terms of version 2 of the GNU General Public License as published by the Free Software Foundation.

SAMMate is distributed in the hope that it will be useful, but WITHOUT ANY WARRANTY; without even the implied warranty of MERCHANTABILITY or FITNESS FOR A PARTICULAR PURPOSE. See the GNU General Public License for more details.

A copy of version 2 of the GNU General Public License is appended in the installation package. For more information, see <http://www.gnu.org/licenses/>.

Table of Contents

Chapter 1 Introduction....4
Chapter 2 Installation....5
2.1. Preparing to Install....6
2.1.1. System Requirements....6
2.1.2. Running Environment....6
2.2 Installing SAMMate....7
Chapter 3 Usages....8
3.1. File Management....9
3.1.1. Working Directory....9
3.1.2. Work Space....10
3.2. SAM/BAM Format Conversion....11
3.2.1. Format Conversion....11
3.3. SAM/BAM File Sorting....12
3.3.1 Sorting a SAM/BAM File....12
3.4. Gene Expression Abundance Score Calculation....13
3.4.1 Processing a Genome Annotation File and RNA-seq Data Files....13
3.5. File Format Customization....15
3.5.1 Customizing a genome annotation file....15
3.5.2 Customizing the signal map intervals....15
3.5.3 Customizing the chromosome names....16
3.6. Memory Configuration on MAC OS....17
3.6.1 Allocating more memory on the MAC OS....17
Chapter 4 Case Studies....18
4.1. Calculating Genomic Feature Abundance Scores....19
4.1.1 Example....19
4.2. Generating a Signal Map for Peak Detection....21
4.2.1 Example....21
4.3. Generating Wiggle File for Visualization....23
4.3.1 Example....23
4.4. Generating Alignment Report....25
4.4.1 Example....25

Chapter 1 Introduction

Welcome to the *SAMMate* Manual.

Here you can find information on how to install and configure SAMMate. It is a step-by-step, task-oriented guide for configuring *SAMMate* on your system.

This manual assumes you have a basic understanding of your operating system. Some installation details are covered in Chapter 2: Installation. If you need detailed instructions on using *SAMMate*, please refer to Chapter 3: Usages and Chapters 4: Use Case Studies.

An HTML version of the manual is available online at SAMMate's homepage:

http://SAMMate.sourceforge.net/

Chapter 2 Installation

This chapter provides a quick overview on installing *SAMMate*.

2.1. Preparing to Install

This section explains SAMMate's requirements.

2.1.1. System Requirements

Recommended Memory: 2GB RAM
Minimum Memory: 1GB RAM
OS: Windows 7, Vista, Mac OS 9, Mac OS X
Note: SAMMate for WinXP can be downloaded from here

2.1.2. Running Environment

A recent version of the Java Runtime Environment (JRE) is needed prior to using SAMMate.

For Windows this involves a quick download from the following Oracle site:
http://www.java.com/en/download/manual.jsp

Java is standard on Mac OS X. For Mac OS 9, you may need to get the Mac OS Runtime for Java (MRJ) from the following Apple site:
http://docs.info.apple.com/article.html?artnum=75097

2.2 Installing SAMMate

- Download the zip file *SAMMate2.4.zip* that matches your OS to your local hard drive.
- Decompress it, and open the software folder *SAMMate 2.4*.
- Double click the executable file *SAMMate.exe* (Windows) or *SAMMate.app* (Mac).

Chapter 3 Usages

This chapter provides a detailed guide of using *SAMMate*. Some typical usage scenarios are listed below:

- Working directory management.
- Adding annotation files and data files to the workspace.
- Removing annotation files and data files from the workspace.
- Converting from SAM format to BAM format and vice-versa.
- Sorting SAM/BAM files.
- Enabling/disabling the building of the coverage file and the signal map file.
- Customization of Genome Annotation File
- Customization of Signal Map Intervals
- Customization of Chromosome Names

3.1. File Management

You can change the working directory and add/remove the annotation file or data files to/from the *Work Space* in SAMMate.

3.1.1. Working Directory

Users can change the working directory for managing the annotation file and data files or for outputting the resulting files.

To display the directory dialogue

- Select File > Open from the menu, or click the 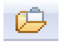 toolbar button.

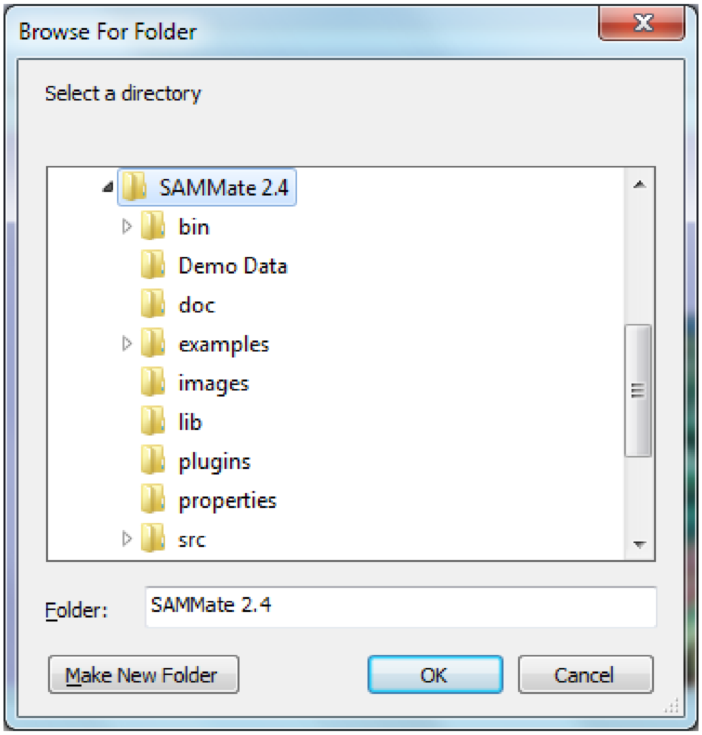

Figure 3.1.1-1 Open directory dialogue

- From the directory tree, select the desired folder, and press the OK button.
- The directory and the files under the selected folder are displayed in the *File Browser Window*.

3.1.2. Work Space

Work Space allows users to add/remove the annotation file and data files.

To add files to the Work Space

- In the *File Browser Window*, right click on the desired annotation or data file, and then left click *Add to Work Space*. Alternatively, double left click on the desired file in the *File Browser Window*. Either method will add the selected file to the *Work Space*.

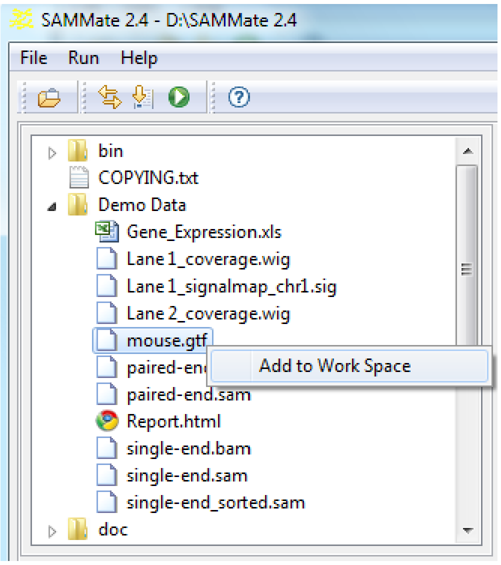

Figure 3.1.2-1 File Browser window

To remove files from the Work Space

- In the *Work Space*, right click on the desired annotation or data file, and then left click *Delete Selection*. Alternatively, double left click on the desired file to remove it from the *Work Space*.

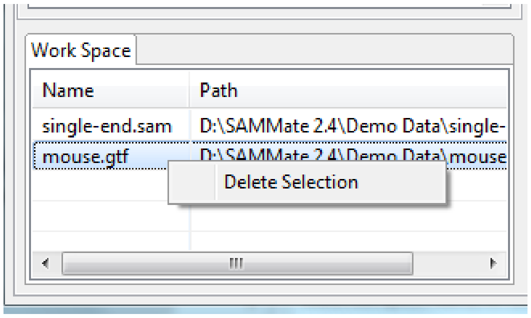

Figure 3.1.2-2 Work Space

3.2. SAM/BAM Format Conversion

SAM is a TAB-delimited text format that is easy to understand, parse, generate and check for errors. However, for extremely large file sizes, SAM is a bit slow to parse. Binary SAM, i.e. BAM, is often used in many production pipelines for intensive data processing.

3.2.1. Format Conversion

SAMMate allows users to convert a file from the SAM format to the Binary SAM (BAM) format and *vice versa*.

To convert SAM/BAM format

- Select File > Run > Convert between SAM and BAM Format from the menu, or click the 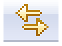 toolbar button.
- A popup dialogue appears with the *Work Space's* SAM/BAM files already loaded.
- By clicking the *Add* button, you can select additional SAM/BAM files to process.
- You can also remove selected SAM/BAM files by clicking the *Delete* button.
- Press the OK button to convert the SAM/BAM files present in the table.

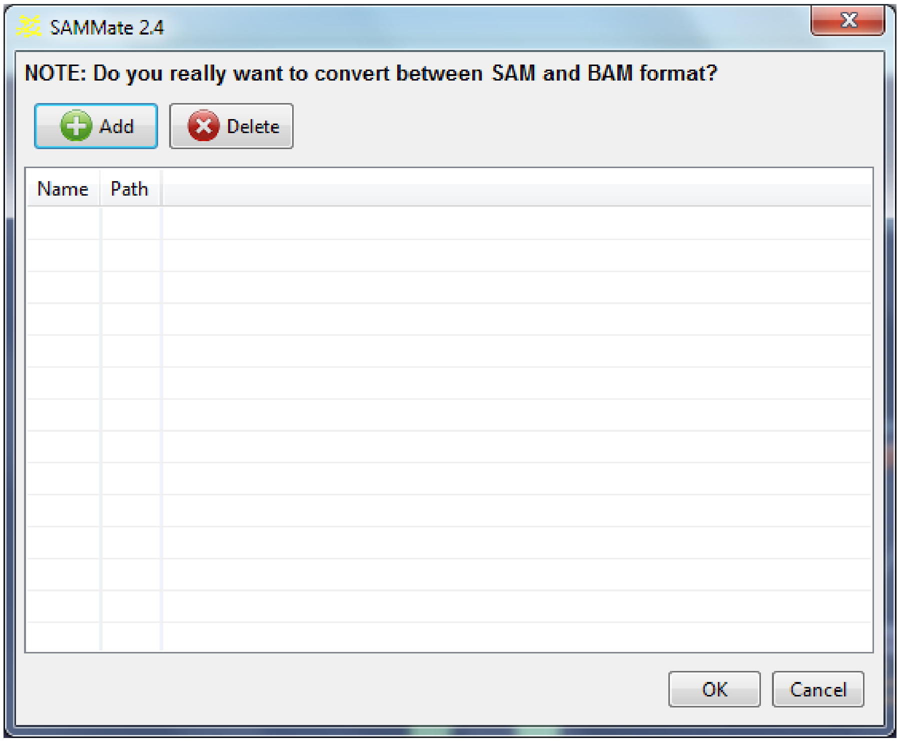

Figure 3.2.1-1 File format converter dialogue

3.3. SAM/BAM File Sorting

A SAM/BAM file can be sorted by the reference coordinates, by query names, or unsorted. Sorting the SAM/BAM file is a crucial step for data processing on a stream and for indexing.

3.3.1 Sorting a SAM/BAM File

SAMMate allows users to sort data files in SAM format or Binary SAM (BAM) format.

To sort a SAM/BAM file

- Select File > Run > Sort SAM/BAM File from the menu, or click the toolbar button.
- A popup dialogue appears with the *Work Space's* SAM/BAM files already loaded.
- By clicking the *Add* button, you can select additional SAM/BAM files to process.
- You can also remove selected SAM/BAM files by clicking the *Delete* button.
- You can check the desired order of sorting (located under the *Add* button).
- Press the OK button to sort the SAM/BAM files present in the table.

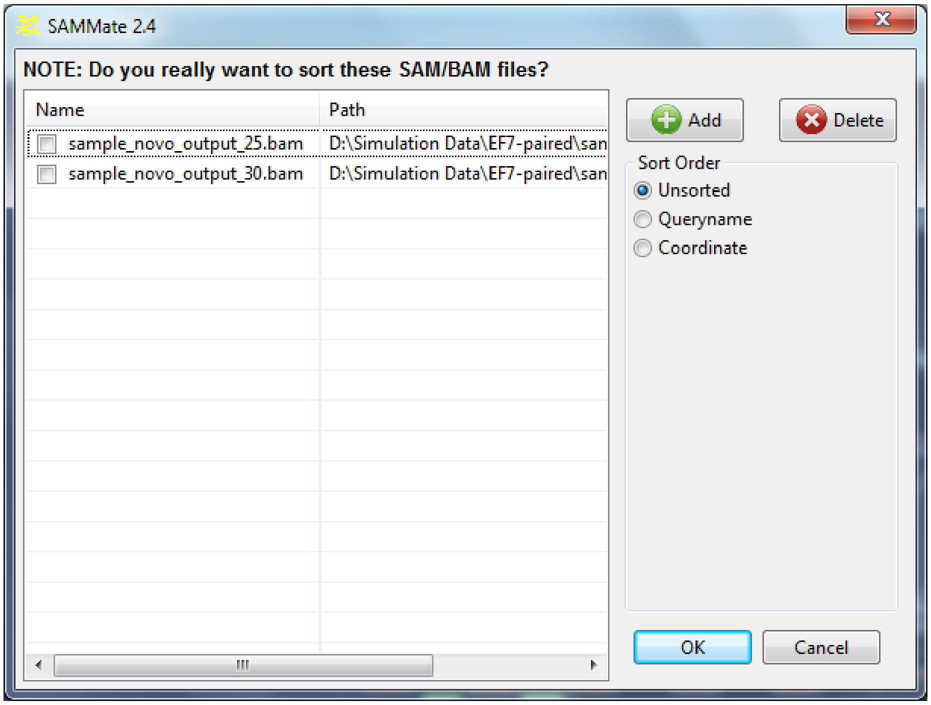

Figure 3.3.1-1 SAM/BAM file sorting dialogue

3.4. Gene Expression Abundance Score Calculation

Using the standard reference genome annotation files, *SAMMate* allows users to accurately calculate the gene expression abundance scores for all annotated genes using RNA-seq data.

3.4.1 Processing a Genome Annotation File and RNA-seq Data Files

SAMMate is able to use short reads originating from both exons and exon-exon junctions to accurately calculate gene expression scores, build coverage depth and generate the signal map for peak detection.

To calculate the gene expression abundance score

- Select File > Run > Run from the menu, or click the 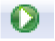 toolbar button.
- A popup dialogue appears with the *Work Space's* SAM/BAM and BED files already loaded.
- By clicking the *Add* button, you can select additional SAM/BAM and BED files to process. You can also add a genome annotation file via the *Add* button.
- You can remove any file in the table by pressing the *Delete* button.
- Set the *User Properties* to enable/disable building a coverage file or signal map file.
- Users can also customize the output f*ile names.*

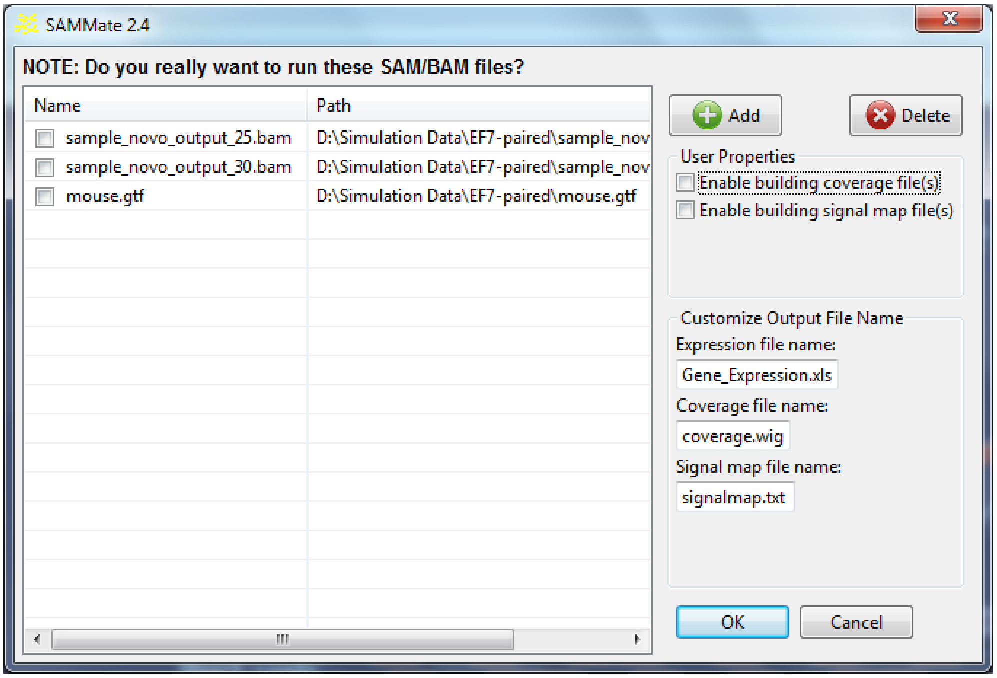

Figure 3.4.1-1 Annotation file and RNA-seq data processing dialogue

- Press the OK button to process the selected genome annotation file and RNA-seq data files in the table.

The selected files ***must*** include only one genome annotation file and at least one SAM/BAM file. The BED file is optional.

3.5. File Format Customization

3.5.1 Customizing a genome annotation file

SAMMate also calculates the abundance scores for customized genomic intervals by customizing a genome annotation file.

- Go to the *SAMMate 2.4* > *example* > *Annotation Files* folder and open one genome annotation file as the reference.
- Follow the format in the selected annotation file to customize a genome annotation file by adding the gene name, chromosome name, exon count, exon start position and end position, etc.
- Add the customized genome annotation file and RNA-seq data files into the Work Space to calculate the gene expression abundance score, coverage depth, signal map and so on.

#geneName	name	chrom	strand	txStart	txEnd	cdsStart	cdsEnd	exonCount	exonStarts	exonEnds
FAM138F	NR_026820	chr1	-	34611	36081	36081	36081	3	34611,35276,35720,	35174,35481,36081,
FAM138A	NR_026818	chr1	-	34611	36081	36081	36081	3	34611,35276,35720,	35174,35481,36081,
FAM138C	NR_026822	chr1	-	34611	36081	36081	36081	3	34611,35276,35720,	35174,35481,36081,
OR4F5	NM_001005484	chr1	+	69090	70008	69090	70008	1	69090,	70008,
LOC100132287	NR_028322	chr1	+	323891	328580	328580	328580	3	323891,324287,324438,	324060,324345,328580,
LOC100132062	NR_028325	chr1	+	323891	328580	328580	328580	3	323891,324287,324438,	324060,324345,328580,
OR4F29	NM_001005221	chr1	+	367658	368595	367658	368595	1	367658,	368595,
OR4F3	NM_001005224	chr1	+	367658	368595	367658	368595	1	367658,	368595,
OR4F16	NM_001005277	chr1	+	367658	368595	367658	368595	1	367658,	368595,
OR4F29	NM_001005221	chr1	-	621097	622034	621097	622034	1	621097,	622034,
OR4F3	NM_001005224	chr1	-	621097	622034	621097	622034	1	621097,	622034,
OR4F16	NM_001005277	chr1	-	621097	622034	621097	622034	1	621097,	622034,

Figure 3.5.1-1 Customizing genome annotation file

3.5.2 Customizing the signal map intervals

SAMMate allows user to customize the signal map intervals to generate the base-wise signal map information that fall between the customized the intervals for peak detection.

- Go to the *SAMMate 2.4* > *properties* folder and open *signalMap.txt*.
- Follow the format in the example file to customize the signal map intervals by adding the chromosome name, start position and end position. See Figure 3.5.2-1.
- Select File > Run > Run from the menu, or click the toolbar button.
- Set the *User Properties* to *enable build signal map file*.
- Press the OK button to build the signal map file.

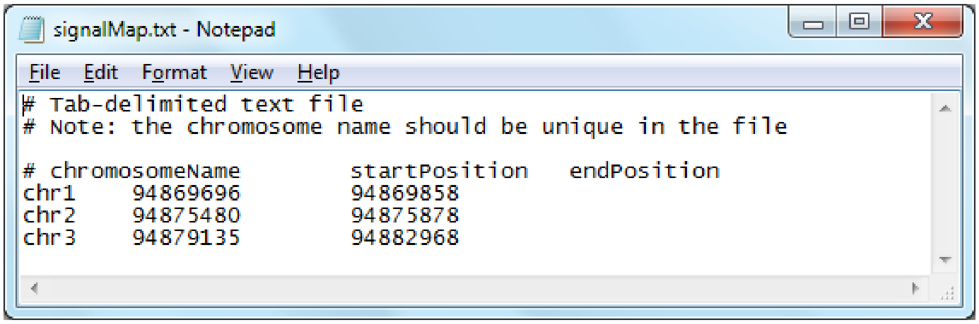

signalMap.txt - Notepad

File Edit Format View Help

```
# Tab-delimited text file
# Note: the chromosome name should be unique in the file

# chromosomeName         startPosition    endPosition
chr1     94869696        94869858
chr2     94875480        94875878
chr3     94879135        94882968
```

Figure 3.5.2-1 Signal map configuration file

3.5.3 Customizing the chromosome names

Between the genome annotation file and the RNA-seq data file, the chromosome names are often mismatched due to different databases and/or aligners. To remedy this situation, *SAMMate* allows user to customize the relationship map between different chromosome names allowing the system to automatically map the customized chromosome names during calculations.

- Go to the *SAMMate 2.4 > properties* folder and open *chromosomesMap.txt*.
- Define the mapping relationship of chromosome names between different versions. For example, by adding add the line:

gi|89161185|ref|NC_000001.9|NC_000001   chr1

SAMMate will automatically replace the string "gi|89161185|ref|NC_000001.9|NC_000001" with "chr1" in the output files.

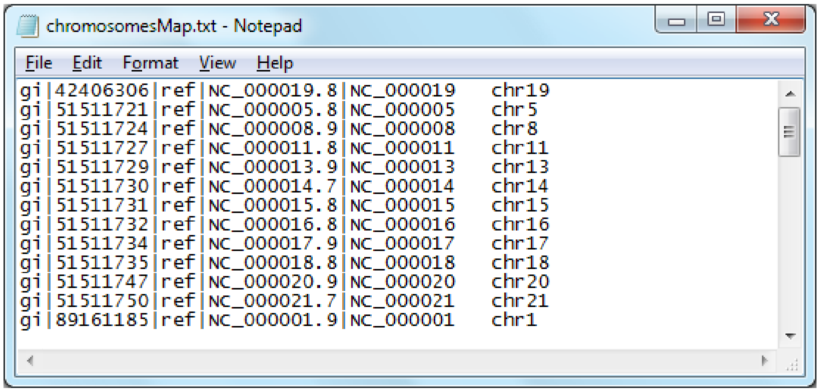

chromosomesMap.txt - Notepad

File Edit Format View Help

```
gi|42406306|ref|NC_000019.8|NC_000019    chr19
gi|51511721|ref|NC_000005.8|NC_000005    chr5
gi|51511724|ref|NC_000008.9|NC_000008    chr8
gi|51511727|ref|NC_000011.8|NC_000011    chr11
gi|51511729|ref|NC_000013.9|NC_000013    chr13
gi|51511730|ref|NC_000014.7|NC_000014    chr14
gi|51511731|ref|NC_000015.8|NC_000015    chr15
gi|51511732|ref|NC_000016.8|NC_000016    chr16
gi|51511734|ref|NC_000017.9|NC_000017    chr17
gi|51511735|ref|NC_000018.8|NC_000018    chr18
gi|51511747|ref|NC_000020.9|NC_000020    chr20
gi|51511750|ref|NC_000021.7|NC_000021    chr21
gi|89161185|ref|NC_000001.9|NC_000001    chr1
```

Figure 3.5.3-1 Chromosomes map configuration file

3.6. Memory Configuration on MAC OS

3.6.1 Allocating more memory on the MAC OS

SAMMate also allows users to increase the amount of memory allocated to improve its performance on the Mac OS platform. Users can easily increase the amount of memory to use by modifying the launching file (*SAMMate.app*).

- Go to the *SAMMate 2.4* folder, and right click on the launching file *SAMMate.app*.
- Select "*Show Package Contents*".
- Go to the folder *Contents*.
- Open the file "*Info.plist*" by double clicking on it.
- In the line Java -> VMOptions, change the parameter from "-Xmx1024M" to "-Xmx2048M".
- Save the changes, and launch *SAMMate.app* again.

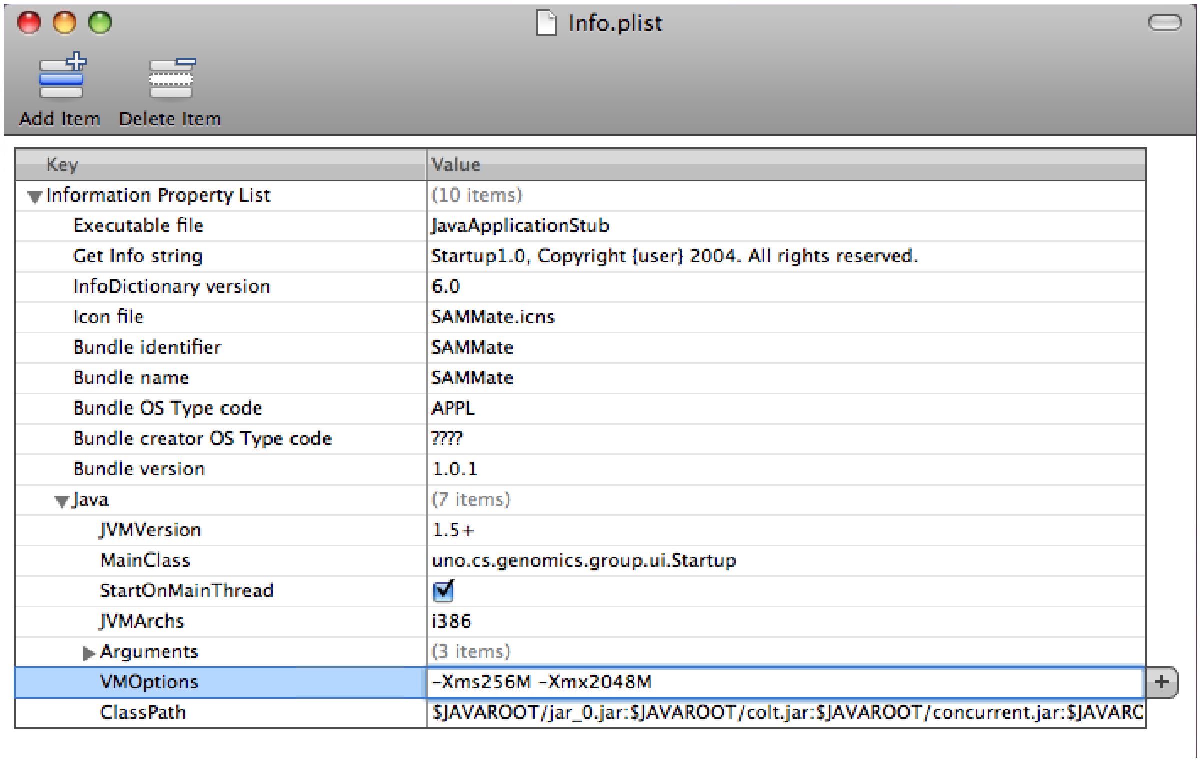

Figure 3.6.1-1 Info.plist file

On a 32-bit machine, the allocated memory cannot exceed 2048MB (or 2GB).

Chapter 4 Case Studies

This chapter provides some detailed examples for user case studies:

- Calculating genomic feature abundance scores
- Generating a signal map for peak detection
- Generating coverage wiggle files for visualization
- Generating an alignment report

The genome annotation file and simulation RNA-seq data files used in these examples can be downloaded from

http://SAMMate.sourceforge.net/download.html

4.1. Calculating Genomic Feature Abundance Scores

SAMMate combines both short reads mapped to exons (e.g. available in SAM/BAM format) and to exon-exon junctions (e.g. available in BED format) to accurately estimate gene expression scores. *SAMMate* can also process many pairs of SAM (BAM)/BED files simultaneously.

SAMMate allows users to calculate the genomic feature abundance scores for any user-defined genomic intervals. This utility dramatically simplifies the technical barriers for discovering novel genes.

4.1.1 Example

Download the genome annotation file and the simulation RNA-seq data to a local hard drive from http://SAMMate.sourceforge.net/download.html. Unzip to a temporary folder, and perform the following:

- Change the working directory to the temporary folder.
- Select File > Run > Run from the menu, or click the toolbar button.
- In the popup dialogue, press the *Add* button to add the genome annotation file, SAM/BAM files or BED files into the table.
- In the popup dialogue, set the *User Properties* to enable/disable building a coverage file and/or a signal map file.

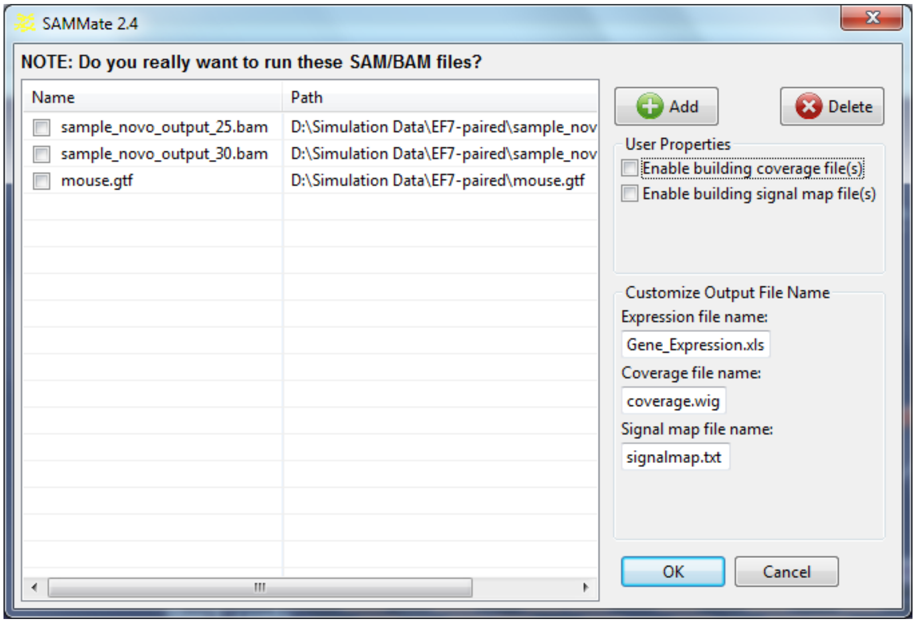

Figure 4.1.1-1 Annotation file and RNA-seq data processing dialogue

- In the popup dialogue, you can customize the output file names.
- Press the OK button to process the selected files.
- After the process is finished, the annotation file and the genomic feature abundance scores are displayed in the *Navigator window*. A Microsoft EXCEL compatible gene expression matrix is also generated in the temporary folder.

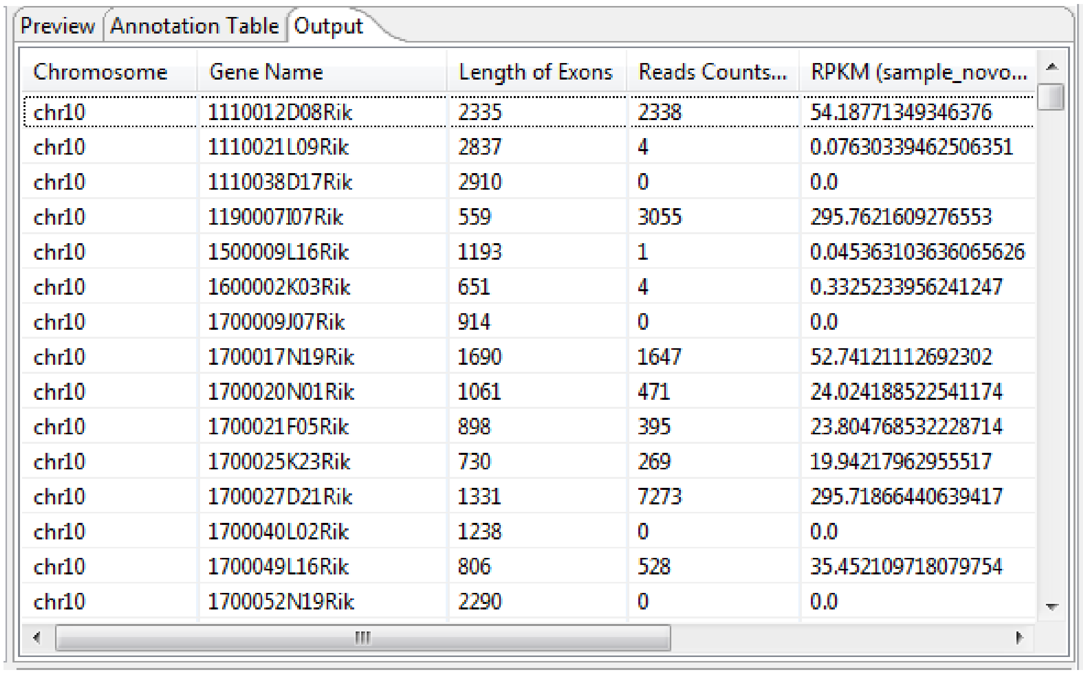

Chromosome	Gene Name	Length of Exons	Reads Counts...	RPKM (sample_novo...
chr10	1110012D08Rik	2335	2338	54.18771349346376
chr10	1110021L09Rik	2837	4	0.07630339462506351
chr10	1110038D17Rik	2910	0	0.0
chr10	1190007I07Rik	559	3055	295.7621609276553
chr10	1500009L16Rik	1193	1	0.045363103636065626
chr10	1600002K03Rik	651	4	0.3325233956241247
chr10	1700009J07Rik	914	0	0.0
chr10	1700017N19Rik	1690	1647	52.74121112692302
chr10	1700020N01Rik	1061	471	24.024188522541174
chr10	1700021F05Rik	898	395	23.804768532228714
chr10	1700025K23Rik	730	269	19.94217962955517
chr10	1700027D21Rik	1331	7273	295.71866440639417
chr10	1700040L02Rik	1238	0	0.0
chr10	1700049L16Rik	806	528	35.452109718079754
chr10	1700052N19Rik	2290	0	0.0

Figure 4.1.1-2 Navigator window

4.2. Generating a Signal Map for Peak Detection

A signal map is also another frequently demanded data format for NGS data analysis. In a signal map file, alignment results are represented in the per-base "pileup" format. In this format the single nucleotide short read coverage depth is calculated whereas the whole genome coverage is provided as a vector of integers with length 3.2×10^9.

SAMMate allows users to generate a signal map for a number of frequently performed sequential analyses to detect a wide range of genomic features based on user-defined genomic intervals.

4.2.1 Example

Download the genome annotation file and the simulation RNA-seq data to a local hard drive from http://SAMMate.sourceforge.net/download.html. Unzip to a temporary folder, and perform the following:

- Change the working directory to the temporary folder.
- Go to the *SAMMate 2.4* > *properties* folder, and open *signalMap.txt*.
- Follow the format in the example file to customize the signal map intervals by adding the chromosome name, start position and end position. Save the file upon completion.

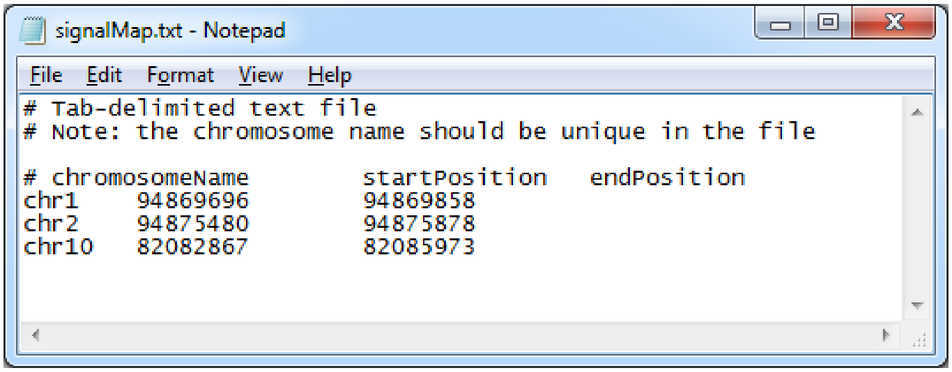

Figure 4.2.1-1 Signal map configuration file

- Select File > Run > Run from the menu, or click the 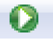 toolbar button.
- In the popup dialogue, press the *Add* button to add the genome annotation file, SAM/BAM files or BED files into the table.
- In the popup dialogue, set the *User Properties* to *enable build signal map file*.

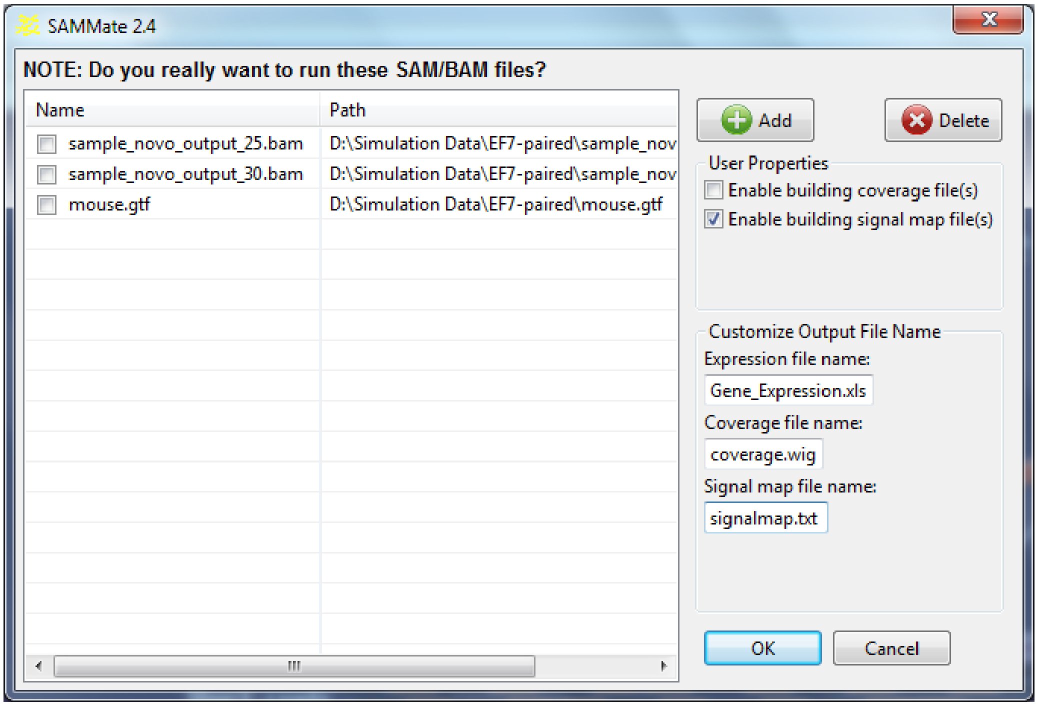

Figure 4.2.1-2 Annotation file and RNA-seq data processing dialogue

- In the popup dialogue, you can customize the output file names.
- Press the OK button to process the selected files.
- After the process is finished, the signal map file ordered by chromosome names is generated in the temporary folder.

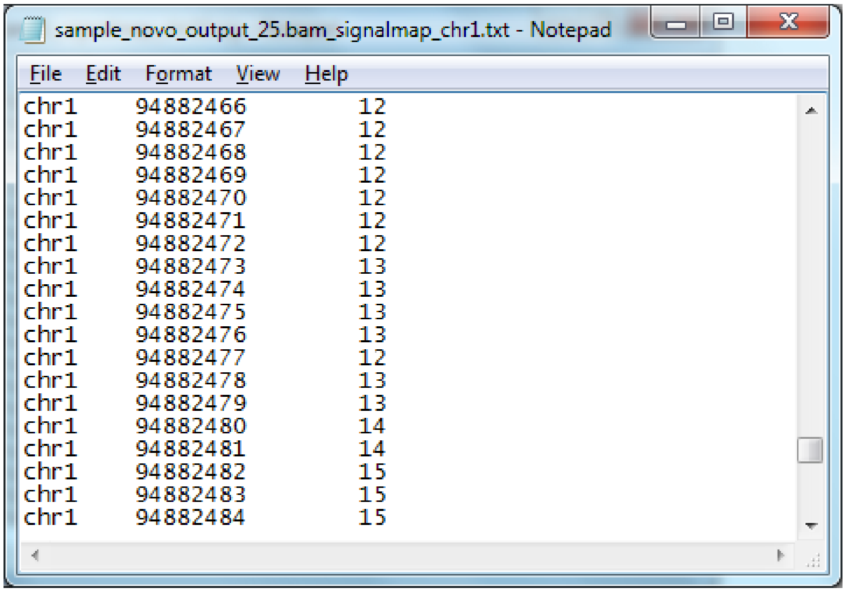

Figure 4.2.1-3 Signal map resulting file

4.3. Generating Wiggle File for Visualization

The wiggle (WIG) format is to display dense, continuous data such as GC percent, probability scores, and transcriptome data. A key *SAMMate* feature is to generate wiggle files for biomedical researchers so that they may visually search for gene structure alterations. These output files are compatible with the UCSC genome browser and other browsers used for visualization. This feature will allow biomedical researchers to visually check the alignment quality of selected genes in the selected genome regions.

4.3.1 Example

Download the genome annotation file and the simulation RNA-seq data to a local hard drive from http://SAMMate.sourceforge.net/download.html. Unzip to a temporary folder, and perform the following:

- Change the working directory to the temporary folder.
- Select File > Run > Run from the menu, or click the toolbar button.
- In the popup dialogue, press the *Add* button to add the genome annotation file, SAM/BAM files or BED files into the table.
- In the popup dialogue, set the *User Properties* to *enable build coverage file*.

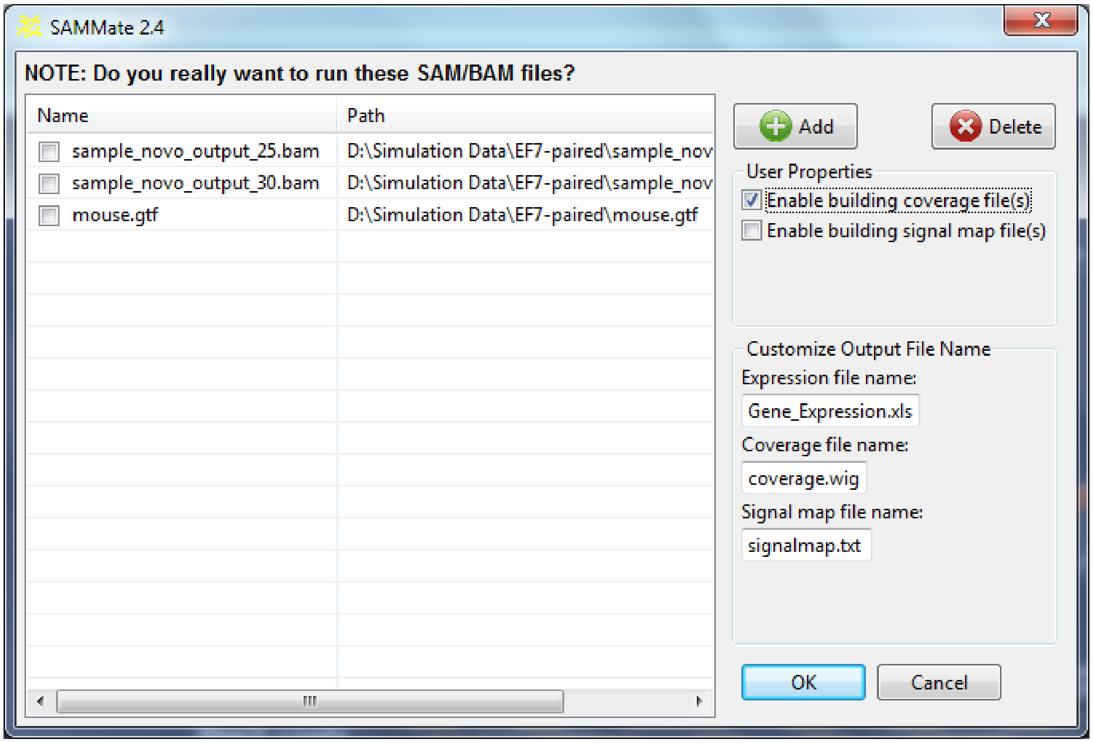

Figure 4.3.1-1 Annotation file and RNA-seq data processing dialogue

- In the popup dialogue, you can customize the output file names.
- Press the OK button to process the selected files.
- After the process is finished, the coverage file sorted by chromosomal position is generated in the temporary folder.

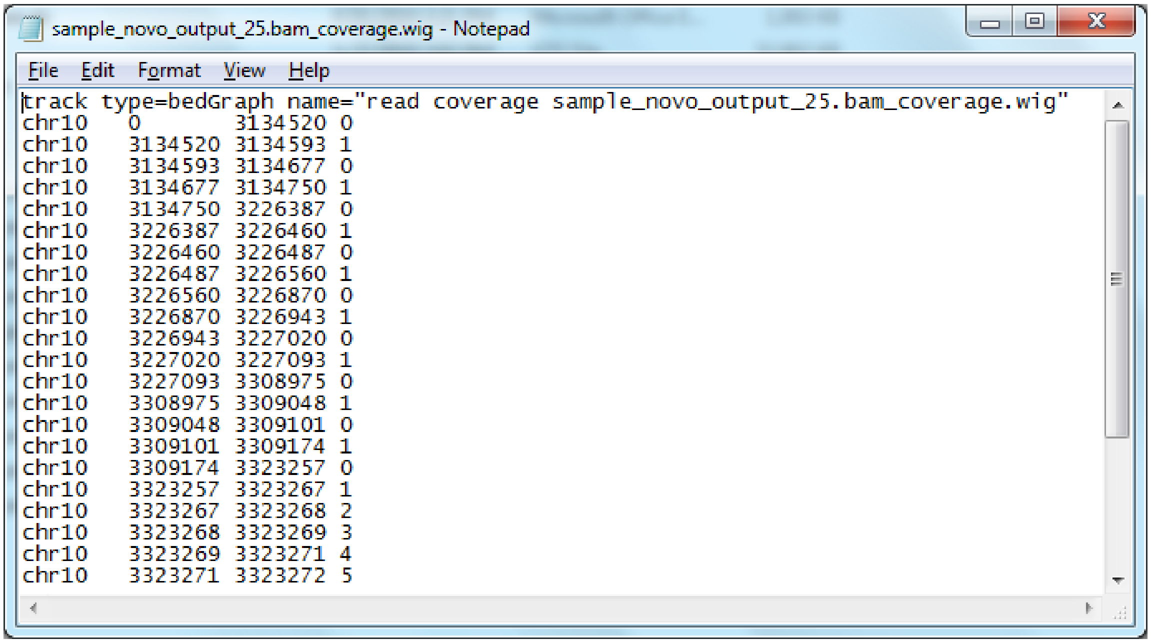

sample_novo_output_25.bam_coverage.wig - Notepad

File Edit Format View Help

```
track type=bedGraph name="read coverage sample_novo_output_25.bam_coverage.wig"
chr10	0	3134520	0
chr10	3134520	3134593	1
chr10	3134593	3134677	0
chr10	3134677	3134750	1
chr10	3134750	3226387	0
chr10	3226387	3226460	1
chr10	3226460	3226487	0
chr10	3226487	3226560	1
chr10	3226560	3226870	0
chr10	3226870	3226943	1
chr10	3226943	3227020	0
chr10	3227020	3227093	1
chr10	3227093	3308975	0
chr10	3308975	3309048	1
chr10	3309048	3309101	0
chr10	3309101	3309174	1
chr10	3309174	3323257	0
chr10	3323257	3323267	1
chr10	3323267	3323268	2
chr10	3323268	3323269	3
chr10	3323269	3323271	4
chr10	3323271	3323272	5
```

Figure 4.3.1-2 Coverage wiggle resulting file

4.4. Generating Alignment Report

Short read alignment statistics provide indispensable resources to examine the alignment quality as well as to compare the alignment results. *SAMMate* calculates and exports a number of alignment statistics including the percentage of uniquely mapped short reads as well as the percentage of short reads mapped to intergenic, exonic and intronic regions.

4.4.1 Example

Download the genome annotation file and the simulation RNA-seq data to a local hard drive from http://SAMMate.sourceforge.net/download.html. Unzip to a temporary folder, and perform the following:

- Change the working directory to the temporary folder.
- Select File > Run > Run from the menu, or click the 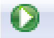 toolbar button.
- In the popup dialogue, press *Add* button to add the genome annotation file, SAM/BAM files or BED files into the table.
- In the popup dialogue, set the *User Properties* to disable build coverage file and signal map file.

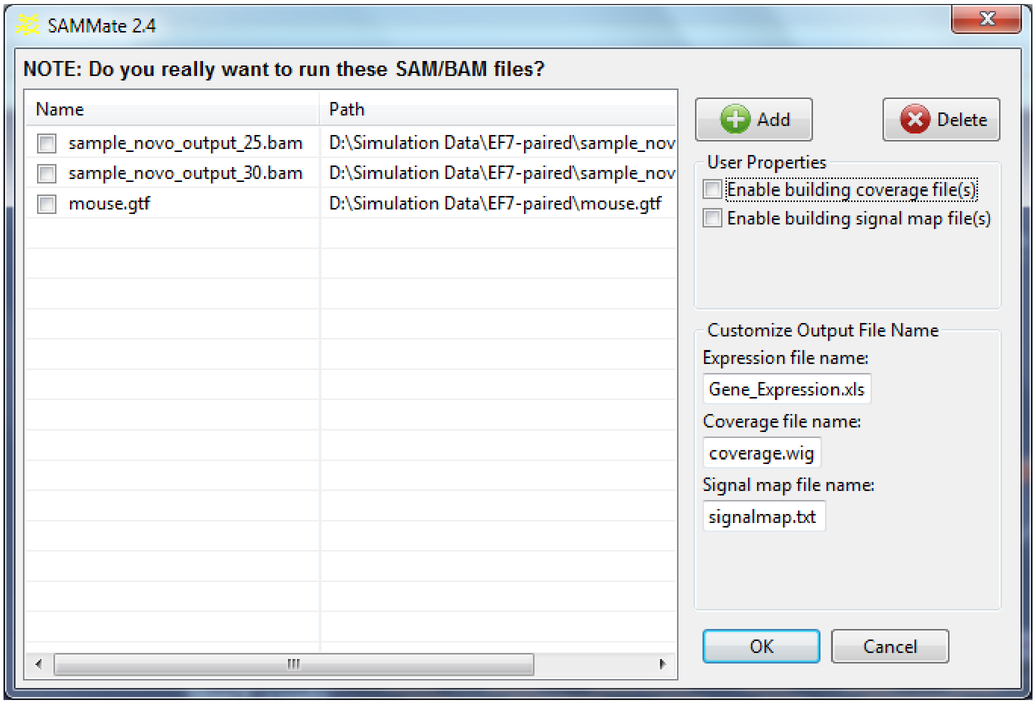

Figure 4.4.1-1 Annotation file and RNA-seq data processing dialogue

- In the popup dialogue, you can customize the output file names.
- Press the OK button to process the selected files.
- After the process is finished, a report file in HTML format is generated in the temporary folder.

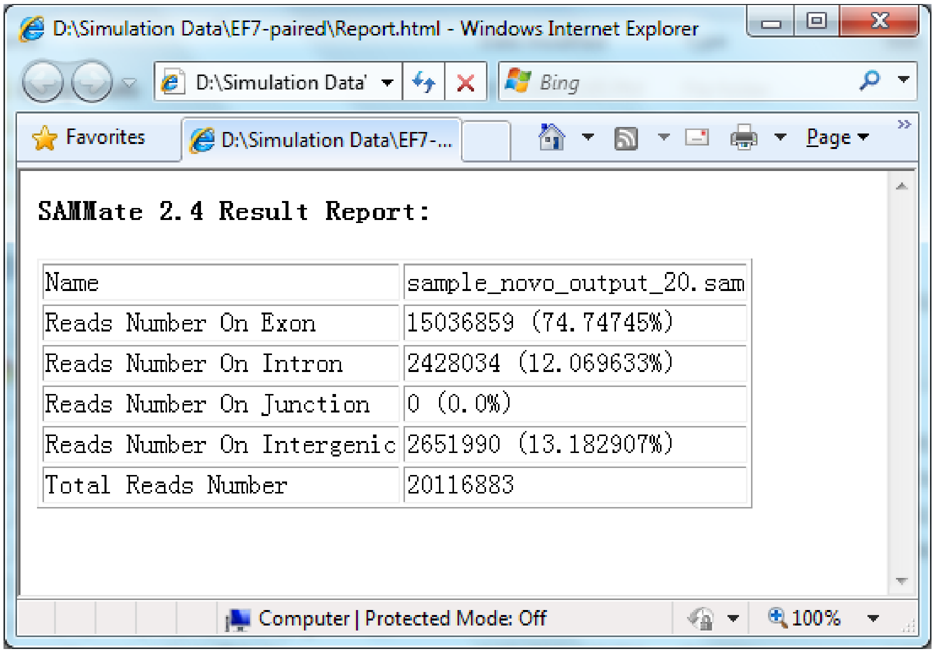

Figure 4.4.1-2 Alignment report file
